# Shed Light in the DaRk LineagES of the Fungal Tree of Life—STRES

**DOI:** 10.3390/life10120362

**Published:** 2020-12-19

**Authors:** Laura Selbmann, Zsigmond Benkő, Claudia Coleine, Sybren de Hoog, Claudio Donati, Irina Druzhinina, Tamás Emri, Cassie L. Ettinger, Amy S. Gladfelter, Anna A. Gorbushina, Igor V. Grigoriev, Martin Grube, Nina Gunde-Cimerman, Zsolt Ákos Karányi, Beatrix Kocsis, Tania Kubressoian, Ida Miklós, Márton Miskei, Lucia Muggia, Trent Northen, Monika Novak-Babič, Christa Pennacchio, Walter P. Pfliegler, Istvàn Pòcsi, Valeria Prigione, Meritxell Riquelme, Nicola Segata, Julia Schumacher, Ekaterina Shelest, Katja Sterflinger, Donatella Tesei, Jana M. U’Ren, Giovanna C. Varese, Xabier Vázquez-Campos, Vania A. Vicente, Emanuel M. Souza, Polona Zalar, Allison K. Walker, Jason E. Stajich

**Affiliations:** 1 Department of Ecological and Biological Sciences, University of Tuscia, 01100 Viterbo, Italy; coleine@unitus.it; 2 Section of Mycology, Italian National Antarctic Museum (MNA), 16121 Genoa, Italy; 3 Department of Molecular Biotechnology and Microbiology, Faculty of Science and Technology, University of Debrecen, 4032 Debrecen, Hungary; benko.zsigmond@science.unideb.hu (Z.B.); emri.tamas@science.unideb.hu (T.E.); kocsis.beatrix@science.unideb.hu (B.K.); pfliegler.valter@science.unideb.hu (W.P.P.); pocsi.istvan@science.unideb.hu (I.P.); 4 Center of Expertise in Mycology of Radboud University Medical Center, Canisius Wilhelmina Hospital, 6532 Nijmegen, The Netherlands; Sybren.deHoog@radboudumc.nl; 5 Fondazione Edmund Mach, 38010 San Michele all’Adige, Italy; claudio.donati@fmach.it; 6 The Key Laboratory of Plant Immunity, Nanjing Agricultural University, Nanjing 210095, China; irina.s.druzhinina@mail.ru; 7 Genome Center, University of California, Davis, CA 95616, USA; clettinger@ucdavis.edu; 8 Microbiology & Plant Pathology, University of California Riverside, Riverside, CA 92521, USA; tkurb001@ucr.edu; 9 Department of Biology, University of North Carolina at Chapel Hill, Chapel Hill, NC 27514, USA; amyglad@unc.edu; 10 Department of Materials and Environment, Bundesanstalt für Materialforschung und -prüfung (BAM), 10115 Berlin, Germany; anna.gorbushina@bam.de (A.A.G.); julia.schumacher@wwu.de (J.S.); 11 Department of Earth Sciences & Department of Biology, Chemistry, Pharmacy, Freie Universität, Berlin 10115 Berlin, Germany; 12 Lawrence Berkeley National Laboratory, US Department of Energy Joint Genome Institute, Berkeley, CA 94720, USA; ivgrigoriev@lbl.gov (I.V.G.); trnorthen@lbl.gov (T.N.); Cppennacchio@lbl.gov (C.P.); 13 Department of Plant and Microbial Biology, University of California Berkeley, Berkeley, CA 94720, USA; 14 Institute of Biology, University of Graz, Graz A-8010, Austria; martin.grube@uni-graz.at; 15 Department Biology, Biotechnical Faculty, University of Ljubljana, 1000 Ljubljana, Slovenia; nina.gunde-cimerman@bf.uni-lj.si (N.G.-C.); monika.novakbabic@bf.uni-lj.si (M.N.-B.); Polona.Zalar@bf.uni-lj.si (P.Z.); 16 Department of Medicine, Faculty of Medicine, University of Debrecen, 4032 Debrecen, Hungary; karanyi.zsolt@med.unideb.hu; 17 Department of Genetics and Applied Microbiology, Institute of Biotechnology, Faculty of Science and Technology, University of Debrecen, 4032 Debrecen, Hungary; miklos.ida@science.unideb.hu; 18 Department of Biochemistry and Molecular Biology, Faculty of Medicine University of Debrecen, 4032 Debrecen, Hungary; miskeim@gmail.com; 19 Department of Life Sciences, University of Trieste, 34121 Trieste, Italy; lmuggia@units.it; 20 Mycotheca Universitatis Taurinensis, University of Torino, 10125 Torino, Italy; valeria.prigione@unito.it (V.P.); cristina.varese@unito.it (G.C.V.); 21 Department of Microbiology, Centro de Investigación Científica y de Educación Superior de Ensenada (CICESE), Baja California 22980, Mexico; riquelme@cicese.mx; 22 Department CIBIO, University of Trento, 38123 Trento, Italy; nicola.segata@unitn.it; 23 Centre for Enzyme Innovation, University of Portsmouth, Portsmouth PO1 2UP, UK; ekaterina.shelest@port.ac.uk; 24 Institute of Natural Sciences and Technology in the Arts, Academy of Fine Arts Vienna, Vienna 22180, Austria; k.sterflinger@akbild.ac.at; 25 Department of Biotechnology, University of Natural Resources and Life Sciences, Vienna 22180, Austria; donatella.tesei@boku.ac.at; 26 Department of Biosystems Engineering and BIO5 Institute, University of Arizona, Tucson, AZ 85721, USA; juren@email.arizona.edu; 27 School of Biotechnology and Biomolecular Sciences, The University of New South Wales, Sydney 2006, Australia; xvazquezc@gmail.com; 28 Department of Biochemistry, Federal University of Paraná, Paraná E3100, Brazil; vaniava63@gmail.com (V.A.V.); souzaem@ufpr.br (E.M.S.); 29 Department of Biology, Acadia University, Wolfville, NS B4P 2R6, Canada; allison.walker@acadiau.ca

**Keywords:** adaptation, black fungi, Dothideomycetes, Eurotiomycetes, extremophiles, genomics, metabolomics, secondary metabolites, stress conditions, transcriptomics

## Abstract

The polyphyletic group of black fungi within the Ascomycota (Arthoniomycetes, Dothideomycetes, and Eurotiomycetes) is ubiquitous in natural and anthropogenic habitats. Partly because of their dark, melanin-based pigmentation, black fungi are resistant to stresses including UV- and ionizing-radiation, heat and desiccation, toxic metals, and organic pollutants. Consequently, they are amongst the most stunning extremophiles and poly-extreme-tolerant organisms on Earth. Even though ca. 60 black fungal genomes have been sequenced to date, [mostly in the family Herpotrichiellaceae (Eurotiomycetes)], the class Dothideomycetes that hosts the largest majority of extremophiles has only been sparsely sampled. By sequencing up to 92 species that will become reference genomes, the “Shed light in The daRk lineagES of the fungal tree of life” (STRES) project will cover a broad collection of black fungal diversity spread throughout the Fungal Tree of Life. Interestingly, the STRES project will focus on mostly unsampled genera that display different ecologies and life-styles (e.g., ant- and lichen-associated fungi, rock-inhabiting fungi, etc.). With a resequencing strategy of 10- to 15-fold depth coverage of up to ~550 strains, numerous new reference genomes will be established. To identify metabolites and functional processes, these new genomic resources will be enriched with metabolomics analyses coupled with transcriptomics experiments on selected species under various stress conditions (salinity, dryness, UV radiation, oligotrophy). The data acquired will serve as a reference and foundation for establishing an encyclopedic database for fungal metagenomics as well as the biology, evolution, and ecology of the fungi in extreme environments.

## 1. Introduction

Fungi are a large group of eukaryotic organisms ranging from unicellular yeasts to multicellular filamentous forms. They have a global distribution due to their small size and their cryptic lifestyle in soil, decomposing matter, and abilities to form a symbiosis with algae, plants, and animals [1,2,3,4]. Fungi are found in every biome including polar, temperate, and tropical environments. Black fungi are an ecologically defined group of stress-tolerant specialists that share morphological similarity despite diverse phylogenetic placement. Black fungi form a polyphyletic morpho-ecological group within Ascomycota, Eurotiomycetes, and “Dothideomyceta” (a clade encompassing Arthoniomycetes and Dothideomycetes) [5]. They are often described with the terms black fungi, black yeasts (BY) and relatives, meristematic fungi, microcolonial fungi (MCF), and rock inhabiting fungi (RIF).

**Figure 1:**
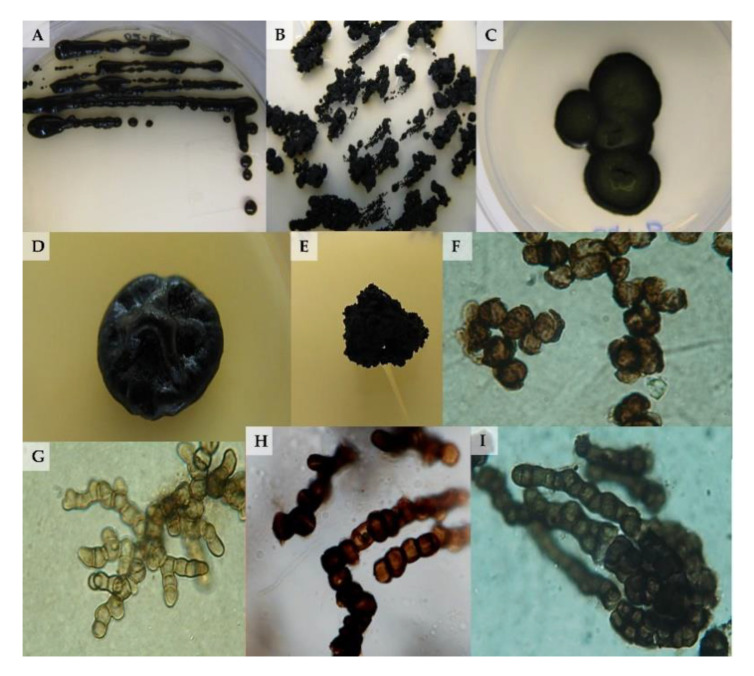
(**A**–**E**) Examples of black fungal colonies grown on Malt Extract Agar plates; (**F**–**I**) micromorphology pictures of black fungi.

A few examples of their morphology are reported in Figure 1.

**Figure 2:**
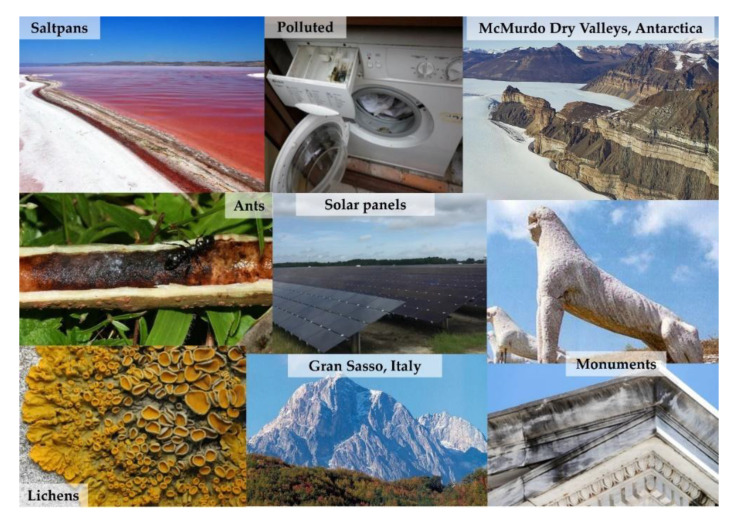
Examples of extreme environments where black fungi have been isolated.

Black yeasts are among the most successful extremophiles and extreme-tolerant organisms on Earth; they are distributed globally in harsh environments that impede colonization by most life-forms. All black yeasts and meristematic fungi share a number of characters, such as yeast-like polar budding, deep melanization, and meristematic growth [6], thick and even multi-layered cell walls, and exo-polysaccharide production, resulting in an extraordinary ability to tolerate chemical and physical stresses. Stresses include extreme pH, high and low temperature, heavy metals, as well as radionuclides, desiccation, high concentrations of different kosmotropic and chaotropic salts [7], UV ionizing radiation, alpha particles, and even real Space and simulated Mars conditions [8,9,10,11,12,13]. They also display a tremendous capacity to resurrect from dry conditions [14]. Constituent melanization and meristematic growth (i.e., conversion towards isodiametric expansion) is infrequent in the fungal kingdom and is a specific response to stress, thus providing the ability to cope with and adapt to highly diverse stressing environments. The black yeasts are also known for their ability to survive in all the extreme habitats including saltpans [15], acidic and hydrocarbon-contaminated sites [16,17,18], exposed natural rocks [19] and stone monument surfaces [20], hot deserts [21], photocatalytic [22] and solar panel [23] surfaces, and very cold icy habitats [24,25,26,27,28,29,30,31]. These fungi can usually colonize human environments like dishwashers, steam baths, or sauna facilities; some have been isolated from a silicone seal in hospitals and in tap water [32,33,34], while other species are domatia-associated [35] (Figure 2). Few of them are involved in a broad range of diseases [36,37], while others, because of their ability to degrade pollutants, are good candidates for bioremediation [38].

To date, black fungi genome sequencing results are only a drop in the ocean and sequences are only available for ca. 60 strains, mainly in the family Herpotrichiellaceae (Eurotiomycetes). In contrast, the class Dothideomycetes which hosts the largest majority of extremophilic black fungi remains largely unsampled. As a result, our understanding of the evolution and adaptation strategies of this intriguing group of fungi remains limited. Studies on the genome evolution of these microorganisms, colonizing a diverse array of inhospitable ecological niches, may enable understanding of important genetic factors that govern their success in the extremes and will provide insights into the existence and the understanding of novel enzymes for keeping an active metabolism under conditions, normally incompatible with [39,40,41].

### Black Fungi Profit from the Era of Genome Consortia

In 1996, the genome of *Saccharomyces cerevisiae* was published and marked the beginning of a new era in fungal biology [42]. Advancements in high throughput sequencing technology have been rapidly progressing and leading to the sequencing of species that can be incorporated into genome-scale phylogenies, as evidenced by MycoCosm [43], with more than 1700 fungal genomes (http://mycocosm.jgi.doe.gov), enabling these data as the starting point for an increasing number and types of researches.

With this rapid development of DNA sequencing technology, this is the time for large-scale, collaborative genomic studies. An international research team in collaboration with the U.S. Department of Energy Joint Genome Institute has embarked on a five-year project to sequence 1000 fungal genomes from across the Fungal Tree of Life (FTOL). The 1000 Fungal Genomes (1KFG) project which started in 2011, aimed to sequence representatives of approximately two genera from each of the roughly 656 recognized families of Fungi [44] and, to date, more than 1500 reference genomes are available [4,45], however, several lineages remain still unexplored. In this era of genome consortia, the overall plan of the “Shed light in The daRk lineagES of the Fungal Tree Of Life” (STRES) project is to fill gaps in the branches of the FTOL, where black yeasts are found to better reveal the genomic traits and fungal metabolites that enable these microorganisms to inhabit and exploit the extremes.

## 2. The STRES Project

STRES (www.stresblackfungi.org) is a 3-year large-scale community science program project funded in September 2019 by the U.S. Department of Energy (DOE) Joint Genome Institute (JGI).

**Figure 3:**
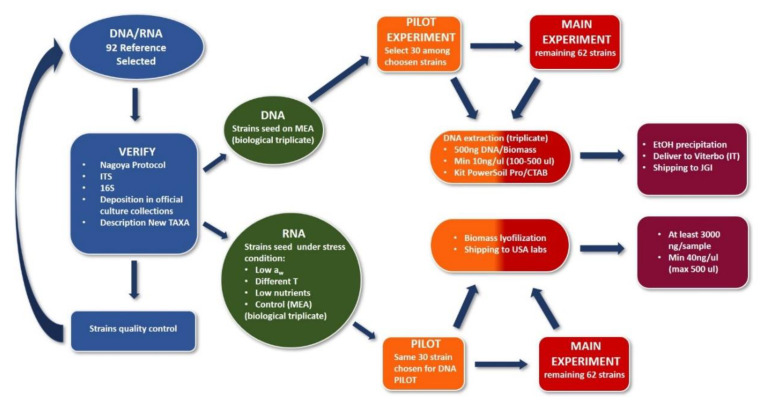
The integrated workflow of the STRES project’s multi-omics challenges is addressed for the construction of high-quality reference genomes.

The STRES project will cover as best the amplitude of black fungal biodiversity along the FTOL by sequencing up to 92 strains as reference genomes, representing primarily unsampled genera, from different ecologies and life-styles (e.g., ant- and lichen-associated fungi, rock-inhabiting fungi, etc.), as well as more than 500 additional strains of black yeasts. We also proposed transcriptomics and metabolomics experiments on a selection of reference species to track transcripts and expressed genes under different stress conditions (i.e., salinity, dryness, UV radiation, and oligotrophy) to further discern their roles in nutrient cycling, interactions in the environment, and to investigate the role of melanin in utilizing radiation as an energy source. The project workflow is outlined in Figure 3.

The STRES consortium is comprised of mycologists, molecular biologists and bioinformaticians from nineteen universities and research institutions mainly from Europe and the US: University of Tuscia (Italy), University of Trieste (Italy), University of Turin (Italy), Fondazione Edmund Mach (Italy), University of Trento (Italy), Center of Expertise in Mycology of Radboud University Medical Center, Nijmegen (The Netherlands), Freie Universität Berlin & Bundesanstalt für Materialforschung und –prüfung, BAM (Germany), University of Ljubljana (Slovenia), University of Natural Resources and Life Sciences (Austria), University of Graz (Austria), University of Debrecen (Hungary), UC Riverside (CA, USA), The University of Arizona (USA), UC Davis (CA, USA), University of North Carolina (USA), Center for Scientific Research and Higher Education of Ensenada CICESE (Mexico), Acadia University (Canada), Nanjing Agricultural University (China), Universidade Federal do Paraná (Brazil), The University of New South Wales Sydney (Australia), and German Centre for Integrative Biodiversity Research (Germany). Furthermore, numerous researchers are actively associated with the project and additional collaborations over the life of the project will be developed.

All strains proposed are currently preserved in private or public culture collections of the international consortium assembled for this project.

The data acquired will serve as a reference and foundation for establishing an encyclopedic database for fungal metagenomics, biology, evolution, and ecology and will further clarify how such fungi adapt and succeed under extreme conditions. These data will also inform on their possible applications in pollutant treatment, as well as possible preventive measures for material protection.

### 2.1. Available Genomic Data

The application of high-throughput sequencing technologies to elucidate the genetic bases of niche adaptation in black fungi started in 2011, when the first whole-genome sequence, belonging to *Exophiala dermatitidis* (Chaetothyriales, Eurotiomycetes, Ascomycota) [46], was sequenced as a part of the Fungal Genome Initiative (http://www.broadinstitute.org/annotation/genome/Black_Yeasts/MultiHome.html). This work was followed by sequencing of four *Aureobasidium pullulans* varieties [47].

Continued efforts generated genomes of additional ca. 50 black fungi, producing an avalanche of data for comparative genomics. We anticipate the genomes of strains proposed in this project will be relatively small (20–50 Mbp) and haploid, with GC content varying between 49–57%, and a very low abundance of repetitive elements. 

In 2013, Lenassi et al. [48] reported the genome of *Hortaea werneckii* (Dothideomycetes) as 51.6 Mb, larger than most phylogenetically related fungi and coding for almost twice the usual number of predicted genes (23k), due to a possible relatively recent whole-genome duplication or hybridization. Gene duplication events might have enabled the rapid evolution of proteins and consequently enhanced the metabolic plasticity, increasing the fitness during the colonization of hostile ecological niches. In 2014, the genome of an Antarctic endolithic black fungus, *Cryomyces antarcticus*, was released for the first time [49]. Several Antarctic cryptoendolithic black fungi (i.e., *Friedmanniomyces endolithicus*, *F. simplex*) have genomes of about 48 Mbp and have a high frequency of gene duplications compared to other extreme-tolerant fungi [50,51]. The analyses of the transcriptome of *Cladophialophora immunda* (Chaetothyriales, Eurotiomycetes), a black fungus typically associated with hydrocarbons polluted environments, revealed that exposure to toluene activated degradation genes, which likely protects the fungus [52]. Teixeira et al. [53] sequenced and annotated 23 *Chaetothyriales* genomes, reporting the genome size varying from 25.81 Mb to 43.03 Mb and identifying a reduction of carbohydrate degrading enzymes. Moreover, some genomes of domatia-associated species showed a relatively small size (ca. 20 Mbp) compared to other Chaetothyriales; it was speculated that, despite the reduction of several protein families, members of the clade might tolerate toxic compounds produced from exocrine glands of the ants as a defense against microbes [35].

### 2.2. Main Objectives

The STRES project has three overarching objectives:

(I) Cover unsampled lineages and ecologies of black fungi.

During the 1st and 2nd years of the project, STRES aims to sequence and make available to the scientific community the whole genomes from 92 black fungal taxa. Fifty-two species in Dothideomycetes, one Arthoniomycetes species, and 39 in the Eurotiomycetes species have been selected as a reference, covering all the main phylogenetic lineages of black fungi. The majority of the selection represents hitherto unsampled groups. Other species will be included to improve their previous poor assembly resolution or because of their very distant phylogenetic relationships with the closest lineages (e.g., *Coniosporium* sp.). Several new taxa have been included and will be described during the project. The selected strains represent diverse ecologies and the breadth of phylogenetic lineages of black fungi for a comprehensive study of evolutionary processes and adaptations of these fungi which could not be undertaken by a single laboratory.

(II) Track transcripts and metabolites under different stress conditions.

Transcriptomics and metabolomics experiments will be performed on a selection of reference species to track transcripts and expressed genes under four different stress conditions (salinity, dryness, UV radiation, oligotrophy) to discern their roles in nutrient cycling, interactions in the environment, and to investigate the role of melanin in utilizing radiation. Transcriptomics and metabolomics experiments will be performed on a selection of reference as the best representative of the main phylogenetic lineages and ecologies: *F. endolithicus*, an endemic species of the Antarctic Desert as the most widespread, and *C. antarcticus* as a recurrent test organism for astrobiological experiments and high multi-stress resistance [9,10,12]. Additional representatives of different ecologies and phylogenies will be sampled among ants- and lichen- associated species, polluted environments, and highly oxidizing surfaces.

(III) Black fungal stress database.

During the 3rd year, a curated repository to provide access to data generated from STRES for comprehensive curated analyses will be developed. Genomics, transcriptomics, and metabolomics will be integrated to look for genes encoding stress response proteins with verified physiological functions and placed in a black fungal stress database. This deep genomic sampling of the diversity of these fungi through the whole genome and transcriptome sequencing will be an immense and valuable resource to understand the organization, regulation, and evolution of stress response systems on black fungi as the background of all major fungal phyla.

### 2.3. Sampling to Sequencing

Sampling has been designed and performed in consultation with all the members of the consortium and will leverage existing biological resources and expertise present in both internationally recognized and private culture collections available for the STRES project.

**Figure 4:**
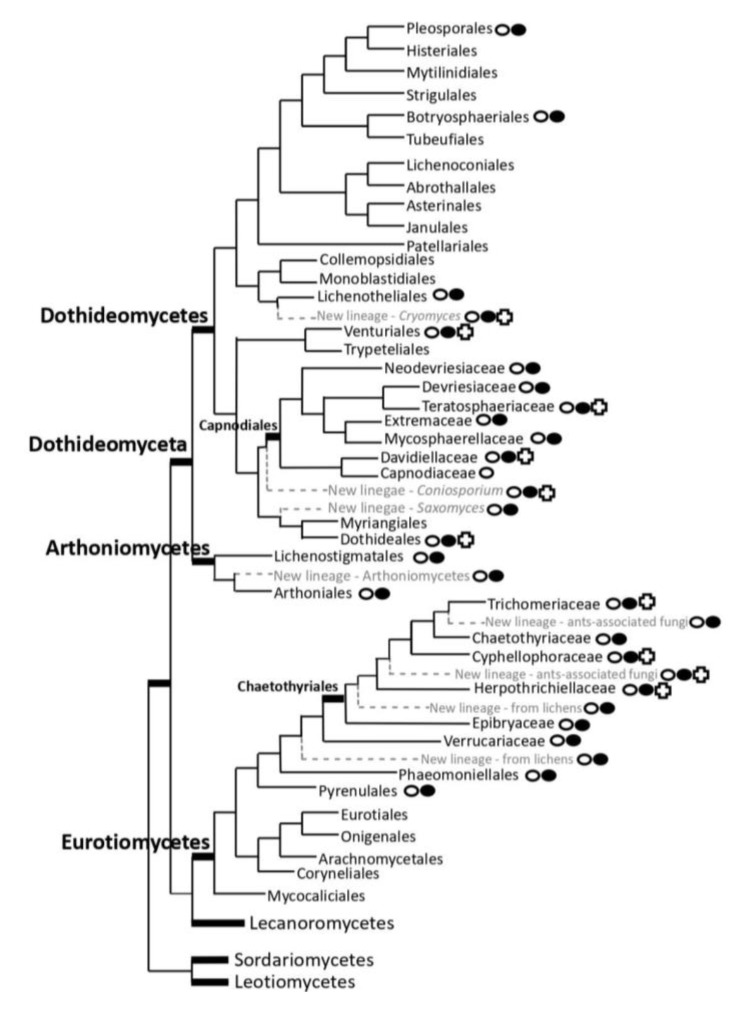
Schematic representation of major lineages in which black fungi are found 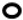 and lineages represented in the selection proposed 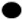 in the ‘STRES’ project; lineages where black fungal genomes of some genera are available 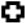.

Selection of the 92 black fungal species as reference genomes has been developed in concert with existing large-scale genome studies to minimize redundancies, overarching most of the main unsampled phylogenetic lineages where black fungi are placed, resulting in a total of 52 Dothideomycetes, 1 Arthoniomycetes, and 39 Eurotiomycetes (Figure 4).

The Dothideomycetes class encompasses many known extremophiles, such as psychrophilic, acidophilic, and halophilic black fungi. Our selection of species aims to ensure a sample from all described genera, ecologies, and geographic distributions including 31 species belonging to 8 families and 6 orders and 16 representatives of new lineages that are being described. Species selected in this class belong to Capnodiales (Teratosphaeriaceae, Neodevriesiaceae, Teratosphaeriaceae, Mycosphaerellaceae, *incertae sedis*), Venturiales (Sympoventuriaceae), Pleosporales (Dydimosphaeriaceae), Dothideales (Dothioraceae), Botryosphaeriales (Botryosphaeriaceae), Lichenostigmatales (*incertae sedis*); Dothideomycetes *incertae sedis* (e.g., *Cryomyces antarcticus, C. funiculosus, Saxomyces penninicus,* and *Coniosporium* spp.).

A genome will be sampled from a new lineage in Arthoniomycetes which is sister to Dothideomycetes and the largest taxonomic group of primarily lichenized fungi outside of Lecanoromycetes.

In the Eurotiomycetes Class, 21 described species belonging to 7 families and 3 orders and 18 representatives of new lineages will be sequenced: e.g., Chaetothyriales (Trichomeriaceae, Herpotrichiellaceae, Cyphellophoraceae, Epibryaceae, Chaetothyriaceae); Chaetothyriales *incertae sedis* including *Phaeoannellomyces elegans* and a species belonging to *Phaeococcomyces;* Verrucariales (Verrucariaceae); Verrucariales *incertae sedis;* Phaeomoniellales (Phaeomoniellaceae).

Furthermore, 550 strains (within ~95% nucleotide identity of reference genomes) will be re-sequenced to identify single nucleotide polymorphisms (SNPs) and characterize intraspecific genomic variability related to specific stress adaptation, geography, and ecology. Most of the 550 strains will be selected from culture collections involved in the project, but additional taxa proposed by international specialists or scientists interested in joining the consortium may be evaluated by the consortium and JGI and eventually included in the project.

### 2.4. Methodologies

Here, the methodologies summarized in the workflow reported above in Figure 3 are here briefly described. We will apply DNA and RNA following community protocols for high purity (e.g., https://dx.doi.org/10.17504/protocols.io.rzkd74w). For the 92 standard coverage genomes, we will provide high-quality DNA/RNA and a proper nucleic acid quantification for Illumina sequencing. The short insert library alone, with standard coverage, has been demonstrated to be more than sufficient for reference genomes, as reported by previous experience (e.g., 1KFG project). We will use an Illumina low coverage-resequencing for up to 550 additional strains within ~95% nucleotide identity with reference genomes.

The STRES project will be able to address critical evolutionary and biological research questions by applying effective analysis methods.

(I) Description of particular genes as hallmarks for the whole group of black fungi.

- *Phylogenomic profiling* to give insights into the evolutionary history of uncovered clades throughout the FTOL (e.g., the origin of symbioses).
- *Single-nucleotide polymorphisms (SNPs) calling* to identify genomic regions contributing to local adaptation or even speciation.
- Detection of *genes duplication* or *whole genome duplication* as events contributing to the ability to adapt to the extremes.
- *Carbohydrate-active enzymes* (CAZymes), assuming that predicted metabolic competences vary among different groups of black yeasts according to their phylogenetic affiliation and ecology.
- *Hydrocarbon and monoaromatic-active enzymes.* Some black fungi, particularly in the order Chaetothyriales, are well known for their ability to degrade pollutants and hydrocarbons. Understanding the distribution and functionality of these genes will also inform us of their possible applications in bioremediation.
- *Stress-tolerance involved enzymes*. Genes involved in stress responses (e.g., UV and ionizing radiation, osmotic, and thermal stresses) will be characterized.
- *Secondary metabolite biosynthetic pathway genes* as potential contributors to local adaptation.
- *Transcription regulators* (TFs) as drivers of adaptation and speciation.

(II) Transcriptomics, metabolomics, and data integration.

Different stress conditions will be tested on a selection of reference species in a special climate chamber “Environment Emulation System” (http://eq-vibt.boku.ac.at/equipment/extreme-climate-chamber/) (relative humidity up to 10%; oligotrophy; UV radiation and salinity stress) available at BOKU University (Austria). We aim to (i) identify potential common/different metabolic patterns across the different ecologies, and (ii) integrate metabolomic (both polar and non-polar metabolites) and transcriptomic data. We are particularly interested in the role of melanin that enables black fungi to utilize radiation for growing [54]; the utilization of these unconventional sources of energy may play a significant role in conditions of continuous nutrient deficiency.

(III) Black fungi genome database and evolution of the stress response system of black fungi.

The Fungal Stress Response Database (http://internal.med.unideb.hu/fsrd2/?p=consortium) [55,56] and the *Saccharomyces cerevisiae*- and *Aspergillus nidulans*-based stress response databases [57] currently incorporate filamentous fungi and yeasts but do not specifically address stress-adapted species. This existing database will be amended with genomes, transcriptomes, and metabolomes that will be obtained in the frame of the STRES project.

## 3. Future Directions

The STRES project will generate an unprecedented, comprehensive data set of black fungal genomes, allowing us to nearly complete the phylogenomic tree for the dark lineages of the FTOL and, in concert with other projects, fungi in general. A broad research community of fungal systematists, ecologists, and geneticists will benefit from the generated data, i.e., the reference genomes and complementing information on fungal biology (metabolic pathway), ecology, and adaptation to stress conditions and extremes. Furthermore, our results will play a critical role in the fungal metagenomics community by providing a much-needed source of phylogenetically diverse, reference genomes. The application of multi-omics approaches to extreme-tolerant and extremophilic fungi will strengthen an existing community of users and attract interests from industries, enabling new, exploitable biotechnological applications.

Additionally, the black fungi stress database, generated from this project, will integrate physiology, ecological and geographic data with completely sequenced and annotated genomes and will represent, for the first time, a systematic, comprehensive, and detailed overview of the stress response of these microorganisms, aiming to decipher the remarkable stress tolerance of these fungi and to stimulate further research in the field of fungal biology. The data acquired will serve to elucidate the possible role of black fungi both in bioremediation and developing material protection measures for stone monuments and solar panels, but most importantly to understand the balance and functionality of extreme ecosystems and to speculate on how life, for as we know it, can adapt and evolve up to the edge of life.
